# Surgical Club of South West England

**Published:** 1986-06

**Authors:** 


### Surgical Club of South West England

**Meeting in Bristol, October 19th and 20th**
Abstracts of papers

### THE PREVENTION OF SPINAL CORD DAMAGE DURING SURGERY OF THE THORACIC AORTA

**G. Keen**
**Bristol Royal Infirmary, Bristol BS2 8HW**

Despite the greatest care to maintain a distal aortic perfusion following aortic cross clamping in operations for coarctation of the aorta, traumatic rupture of the aorta or for the excision of aortic aneurysms a number of patients will develop paraplegia. The greatest risk is during operations for traumatic rupture of the aorta or for acute dissection of the aorta (when paraplegia may present even before operation) but the greatest tragedy is the development of paraplegia following an uncomplicated operation for coarctation.

The risk during surgery for this condition is about one in every two hundred patients and may occur despite all care to maintain distal perfusion. It is considered that this is associated with an abnormality of blood supplied to the spinal cord making cross clamping of the aorta, even for a short period, hazardous in some patients. There is no way to ascertain such abnormality prior to or during surgery. For this reason we advise that consideration should be given to the use of somato-sensory evoked potentials. Briefly these are signals received at the cerebral cortex (by E.E.G.) by a stimulus to a sensory receptor, such as the skin of the thigh. Should continuous signals be received by E.E.G. during surgery then clearly spinal cord function is good, but should these signals disappear the danger of ischaemic transection of the spinal cord is real.

The routine availability of this investigation during surgery and its interpretation is by no means straightforward but it is inevitable that such monitoring be used if the rare disaster of post-operative paraplegia is to be eliminated.

### OESOPHAGEAL FUNCTION STUDIES BEFORE AND AFTER SURGERY

**K. Jeyasingham**
**Frenchay Hospital, Bristol BS16 1LE**

The oesophageal motility laboratory was established in the Department of Thoracic Surgery at Frenchay Hospital in 1975. Since then an increasing number of patients has been investigated before and after surgery, to assess existing functional disorders and to evaluate the results of surgery. Amongst the tests routinely employed are the standard acid reflux test, prolonged monitoring of pH over 24 hours, estimation of the manometric profile of the oesophagus from the cricopharyngeal sphincter to the lower oesophageal sphincter as studied by the slow pull through and the rapid pull through techniques, and manometric response to swallow both wet and dry.

The paper outlines some of the clinical research projects undertaken in the laboratory.

Patients undergoing the oesophageal lengthening V-Y gastroplasty and partial fundoplication showed a good manometric response to swallow in addition to adequate control of reflux. Comparable groups of patients in whom partial fundoplication had been performed for a reducible hiatal hernia with those in whom a partial fundoplication had been performed after a V-Y oesophageal lengthening gastroplasty for shortened oesophagus, revealed very similar resting manometric profiles and response to swallow. Patients undergoing a modified Heller cardiomyotomy for achalasia of the cardia studied before and after with manometry, pH studies, endoscopy and radiology showed that the results in a long term follow-up were excellent in 80 to 90% of the series. The paper briefly outlines the role of the oesophageal laboratory in the day to day functioning of an active Department of Thoracic Surgery.

### PANCREAS TRANSPLANTATION

**P. McMaster**
**Queen Elizabeth Hospital, Birmingham B15 2TH**

Pancreas transplantation was first performed in man in 1967 by Kelly and Lilihi but overwhelming technical problems and the need to administer steroids to prevent rejection meant, that few pancreatic grafts functioned for longer than one year.

With increasing numbers of diabetics entering renal failure programmes in the seventies, it became apparent that renal transplantation alone would not delay or prevent the progression of the microangiopathic complications. The development of new techniques for pancreatic transplantation involving either ductal injection or enteric drainage coincided with the introduction of newer immunosuppressive agents which were non-steroidal (Cyclosporin A).

Since 1980 there has been a slow but steady improvement in the overall results of pancreatic grafting so that patient mortality has now been reduced significantly and although morbidity may occur after pancreatic implantation, there is no significant inceased risk of patient mortality. From the first series of pancreatic grafts between 1966 and '77 when only 3% of pancreatic grafts functioned at one year, now over 40% of grafts are functioning and in some centres that figure now exceeds 60%.

With the improvement in technique a more realistic appraisement of the role of pancreatic transplantation can be made and may now become sufficiently safe to undertake in diabetic patients with less advanced major diabetic complications.

### THE MANAGEMENT OF AORTO-ENTERIC FISTULAE

**W. E. G. Thomas and R. N. Baird**
**Bristol Royal Infirmary, Bristol BS2 8HW**

Aorto-enteric fistula is a rare but devastating complication of prosthetic reconstruction, carrying a mortality of 45% or more. There is no uniform approach to management, but American authors deem graft excision mandatory, usually accompanied by extra-anatomic revascularisation. This procedure carries a high incidence of aortic stump disruption, and our present experience suggests it may be unnecessary in many cases.

Eight patients (M:F 5:3) (age 14–81) developed aorto-enteric fistulae (1 recurrent) between 9 months and 10 years following prosthetic aortic reconstruction. All presented with gastro-intestinal haemorrhage, often recurrent over periods of 24 hours to 2 months. In all cases a preoperative diagnosis was made on clinical grounds but not before delay due to multiple investigations. At laparotomy 2 grafts surrounded by pus, were excised and one underwent an axillo-bifemoral revascularisation. In the remainder, 4 had a simple suture repair of the defects and 2 had local regrafting. In 1 case adequate graft cover was not achieved and the fistula recurred at 2 months. Repeat closure of the fistula was undertaken with omental interposition. Of the 2 patients who underwent graft excision, 1 died of disseminated sepsis, and the other, a 14 year girl with diffuse aneurysmal disease, died 9 months later of aortic disruption. Those who had simple closure of the fistula or regrafting are all alive and well at 9 months to 4 years.

These results suggest that graft excision is only mandatory for severely infected prostheses, while the majority of fistulae can be successfully managed with simple suture repair.

### SPLENECTOMY FOR THE PHYSICIAN

**H. J. O. White**
**Southmead Hospital, Bristol BS10 5NB**

The outcome of 76 patients undergoing splenectomy for non-traumatic indications at Southmead Hospital between 1968 and 1985 was reviewed, 46 patients being a personal series.

22 patients underwent splenectomy to stage their Hodgkins' disease, which affected the spleen of 7, with one late death. 13 patients, who had histologically normal spleens removed, are alive and well, up to 13 years later. Two are dead, one of recurrence, and one with leukaemia. All 8 patients with non-Hodgkins' lymphoma survived 5 years or more.

Both patients with myelofibrosis died within 6 months. 10 patients underwent splenectomy for leukaemia, 3 with chronic lymphatic disease, 2 dying within a year. All 4 patients with chronic myeloid leukaemia were dead within the year, 2 in the immediate postoperative period with continuing haemorrhage, despite exploration. Both patients with hair cell leukaemia, and 1 with myoblastic disease were alive and well at 5 years. 7 patients with idiopathic thrombocytopenic purpura remain alive and well. All 5 children with congenital spherocytosis and 2 adults out of 3 benefited from this operation. 1 adult died a year later.

There was an immediate postoperative mortality of 4%, and 8% within a year. Sepsis was a serious complication in 2 cases. The need for good access, drainage, early re-exploration for continuing haemorrhage, and close liaison with the referring haematologist was emphasised.

### THE INJURED SPLEEN—IS THERE A PLACE FOR CONSERVATION

**M. J. Cooper and R. C. N. Williamson**
**Bristol Royal Infirmary, Bristol BS2 8HW**

The first total splenectomy for trauma took place in 1678, since which it has become the standard operation for the injured spleen. However, although the spleen is not essential for life it does have haematological and immunological functions, particularly with regard to opsonin production. In 1919 it was discovered that asplenic rats were susceptible to death from infection and this has since been fully documented in man. This increased risk of overwhelming post-splenectomy sepsis and the recognition that active bleeding has often stopped at the time of laparotomy has prompted a new approach to splenic trauma. The alternatives to splenectomy are either to adopt a non-operative approach or to repair or partially resect the injured spleen. Between January 1984 and March 1985 we adopted a non-operative approach in 8 young adults (7M:1F) with a mean age of 22 years, (range 18–28 years) who were admitted following abdominal trauma and had radionuclide or ultrasound scan evidence of splenic injury. All patients responded to initial transfusion and 7 recovered without any further evidence of bleeding. One patient continued to bleed intermittently and underwent laparotomy and hemisplenectomy 5 days after admission. In selected young adults who fulfil Simpson's criteria a conservative (non-operative) approach to splenic injury appears to be safe and successful.

### THE INFLUENCE OF ENDOSCOPIC PAPILLOTOMY ON THE MANAGEMENT OF BILE DUCT STONES

**M. H. Thompson**
**Southmead Hospital, Bristol BS10 5NB**

The present role of endoscopic papillotomy in the management of bile duct stones may be assessed in units where a single team carries out both endoscopic and operative treatment.

In the 4 year period from September 1981, 291 patients were treated for gallstones in a single surgical unit. Of these, 49 (17%) underwent surgical exploration of the common bile duct, and 64 (22%) endoscopic papillotomy. The average ages of these patients were: cholecystectomy alone 51.4, surgical duct exploration 60.8 and endoscopic papillotomy 72. Endoscopic papillotomy was used in preference to surgery when the patient was old, or considered a poor operative risk.

Two elderly patients died, both after operation following failure of endoscopic treatment.

Of the 49 surgical duct explorations, 10 patients had choledochoduodenostomy or sphincteroplasty. Retained stones occured in 4 patients, one requiring re-operation. There was major morbidity in 6 patients.

There were 16 failures to achieve a satisfactory endoscopic papillotomy. Four patients required operation for failure to clear stones endoscopically. The remaining 48 achieved duct clearance with minimal morbidity and good clinical results.

Twenty-three patients (of average age 82) treated endoscopically had intact gall bladders. Fifteen were rendered asymptomatic and one only had continuing gall bladder pain.

These results show that bile duct stones in older patients with gallstones and a large proportion can be treated endoscopically with little morbidity. It may not be necessary to add a cholecystectomy.

### ARE GALLSTONES PREVENTABLE?

**K. W. Heaton**
**Bristol Royal Infirmary, Bristol BS2 8HW**

Gallstones are preventable. The prevalence of gallstones varies markedly in different countries and correlates roughly with the degree of 'westernisation' of a country. Within a country having a high prevalence the strongest risk factors are increasing age, female sex, and parity, but reversible risk factors have also been identified. These are obesity, hypertriglyceridaemia and, probably, high fasting plasma insulin concentrations and low plasma HDL-cholesterol levels. All of these factors are related to diet and, specifically, to excess total calories and to high intakes of sucrose. Case control studies have shown a high sucrose intake to increase the risk of gallstones. Lack of dietary fibre is probably a contributory factor. The biochemical prerequisite for gallstone formation is supersaturation of gallbladder bile with cholesterol. Adding wheat bran to the diet or replacing sugar and white flour with fruit and wholemeal flour lowers the cholesterol saturation of bile. Conversely, when normal people are made constipated by taking the anti-diarrhoeal drug loperamide, their bile becomes more saturated with cholesterol. This is probably because they absorb more deoxycholic acid from their colon, this bile acid being a bacterial metabolite and one which causes the liver to secrete more cholesterol. Gallstones should be prevented by staying slim and by eating a low-sugar, high fibre diet.

### THE CLINICAL SPECTRUM OF LYMPHOID THYROIDITIS

**A. John Webb**
**Bristol Royal Infirmary, Bristol BS2 8HW**

The commonly accepted form and presentation of lymphoid or Hashimoto's thyroiditis is well described in the standard literature and text books. Less well appreciated is the atypical or nodular presentation of this disease. The author's series of aspiration biopsies (FNAB) from 1979–1984 comprised 1367 patients of which 259 were goitres. Within this group were 37 examples of thyroiditis. This sub-group was carefully studied and the majority (27/37) presented atypically.

Scintiscan, ultrasound scanning and antibody titres are imprecise in detecting atypical thyroiditis. Fine needle biopsy is the only reliable means of recognising this often perplexing lesion. It may closely simulate malignancy; regress poorly with thyroxine therapy and be associated with regional lymphadenopathy.

Further studies are in progress, in particular the relation of thyroiditis to non-Hodgkin's lymphoma.

### KIDNEY STONE SURGERY? A SECOND CLASS SERVICE

**J. C. Gingell**
**Southmead Hospital, Bristol BS10 5NB**

The traditional open surgical removal of calculi from the upper urinary tract can no longer be considered to be the best option for the patient. With advances in percutaneous surgery and extracorporeal shock wave lithotripsy (ESWL), it is now possible to deal with the vast majority of stones by these less invasive techniques. It has been estimated that approximately 95% of all renal calculi can be successfully treated by these methods alone or in combination. Most departments of urology have acquired the instrumentation to undertake percutaneous surgery with the necessary radiological co-operation and expertise. ESWL, which was developed in West Germany, is not so readily available to NHS patients. In the UK there is only one referral centre for such patients at St Thomas' Hospital and to date no firm plans for siting further machines other than in the private sector. Considering the prevalence of renal stone disease in this country and the fact that an ESWL machine will service a population of 5 million, then 10 machines would be necessary. The capital outlay is currently 1 million pounds per machine and the running costs are also considerable. Despite this it is calculated to be the most cost-effective treatment available. The patients return to work after treatment and not to a lengthy convalescence. With increasing public awareness of this 'non-operative' treatment it is predictable that increasing pressure will be put on the DHSS to provide this service. From a logistic point of view this should be planned on a regional or supra-regional basis.

### CAN ACUTE RETENTION FOLLOWING ABDOMINAL SURGERY BE PREDICTED BY URINARY PEAK FLOW MEASUREMENT?

**D. A. Gillatt, R. E. May, P. H. Abrams**
**Frenchay Hospital, Bristol BS16 1LE**

Acute urinary retention is a well recognised complication following abdominal surgical procedures. A prospective study was undertaken to assess the use of a detailed urological history, urine culture, urinary peak flow and urine flow volume measurement in predicting which patients will develop acute retention postoperatively.

Sixty-one male patients aged 21 to 82 years undergoing elective abdominal surgery had urinary peak flow measurement using a urine flow monitor (Lectromed type 4981) performed prior to operation. A peak flow rate of less than 15 mls per sec may denote significant obstruction, patients with rates above this level rarely requiring prostatectomy (1). In this study seven of eleven patients, aged 65 to 78 years, with flow rates below 15 mls per sec developed retention after operation. Only two of fifty, with a flow rate above this level developed retention (CHI squared test P=less than 0.001.) Therefore there is a significantly greater chance of retention developing with a flow rate below 15 mls per sec.

These results suggest that urinary peak flow measurement is a useful investigation in the elderly male prior to laparotomy. As all seven patients developing retention with demonstrable outflow obstruction were over 65 years its use should be confined to this group.

### REFERENCE

1. BALL, A. J., FENELEY, R. C. L., ABRAMS, P. H. (1981). The natural history of untreated 'Prostatism'. *Br.J.Urol.* **53**, pp. 613–16.

### MICROVASCULAR SURGERY WITH A REVIEW OF A PERSONAL SERIES OF 75 RECONSTRUCTIVE CASES

**P. L. G. Townsend**
**Frenchay Hospital, Bristol BS16 1LE**

The anastomosis of vessels 1–3 mm in diameter using a microscope is now a regular part of plastic surgery practice, in the acute situation especially as part of a replantation service, more frequently microvascular surgery is used now in reconstructional procedures.

Recently Mr. Waterhouse reviewed some 42 digital replants which were carried out at Frenchay. The most successful being clean cut guillotine amputations, absolute indications for replantation are amputation of the hand, thumb and two or more fingers. It is very important that during transfer of the separated digit, that it should not be frozen but only cooled.

For this presentation, some 75 personal reconstructive microvascular cases were reviewed excluding replants. The majority, 44, were for lower leg mainly post traumatic problems and the rest to other areas of the body.

Some 32 of the lower limb problems were associated with compound fractures of usually the tibia. The association of skin loss with bone loss over 5 cms in length, has previously been associated with a high amputation rate. Of this series, only one case due to a failed anastomosis had to have his leg amputated, the majority with bone defects 5–10 cms in length were reconstructed with

*(Continued on page 63)*

### Surgical Club of S. W. England *(Continued from page 71)*

vascularised skin and bone (deep circumflex iliac flap) achieving full weight bearing with clinical and radiological union by six months.

Bone defects longer than 10 cms required insertion of a vascularised fibula; over about eighteen months, this undergoes hypertrophy if stressed. Vascularised fibula can also be used in cases of congenital pseudoarthrosis and avascular necrosis.

Other microvascular reconstructional techniques demonstrated included vascularised nerves, tendons, jejunum and finally testicular transfer.

